# The role of macrocyclic compounds in the extraction and possible separation of platinum and rhodium from chloride solutions

**DOI:** 10.1038/srep27668

**Published:** 2016-06-10

**Authors:** Rajesh Kumar Jyothi, Jin-Young Lee

**Affiliations:** 1 Strategic Minerals Utilization Research Department, Convergence Research Center for Development of Mineral Resources (DMR), Korea Institute of Geoscience and Mineral Resources (KIGAM), Daejeon 305-350, Korea

## Abstract

Macrocyclic compounds (crown ethers), specifically 18-crown-6 (18-C-6), benzo-15-crown-5 (B-15-C-5), di-benzo-18-crown-6 (DB-18-C-6) and di-cyclohexano-18-crown-6 (DC-18C-6), are used as extractants as well as synergists with amine-group extractants. Platinum and rhodium belong to platinum-group metals (PGMs) and have very similar ionic radii and similar properties. The separation of PGMs is most useful for the preparation of functional materials. Macrocyclic compounds are tested for platinum and rhodium separation and are found to achieve marginal separation. Amines (used as extractants) are paired with macrocyclic compounds (used as synergists), and the separation factor between platinum and rhodium is increased with synergistic enhancement from a chloride solution. The present study discusses extraction chemistry, separation factors and the synergy between platinum and rhodium from chloride solutions. To ensure accurate data, the aqueous samples in this study are analyzed using an inductively coupled plasma optical emission spectrometer (ICP-OES).

Macrocyclic complexes exist in various categories, and among these, crown-type macrocyclic compounds are known as “crown ethers.” Macrocyclic polyethers were discovered by the American scientist Charles Pederson in 1967^1^,^2^,^3^ and were initially reported in the Journal of the American Chemical Society. In his study, he described the reaction between catechol and bis(2-cholro-ethyl) ether in the presence of NaOH to form 2,3-benzo-1,4,7-tri-oxacyclononane. This study revealed that this crown ether (CE) was an 18-membered ring CE, i.e., di-benzo-18-crown-6.

The three chemical parameters of the permanent dipole moment, polarizability, and Van der Waals radius of a ligand atom influence the interaction selectivity of the ligand to a metal ion. Data indicate that an increase in the dipole movement and/or the polarizability decreases in the radius of the ligand, possibly increasing the stability of hypothetical complexes as well^4^.

In the literature, very few attempts have been made to describe the synergistic extraction of platinum and rhodium.

The two extractants of 8-hydroxyquinoline (HQ) and tributylphosphite (TBPI) were mixed and utilized for platinum and palladium extraction. HQ and its derivatives, in this case 2-methyl-HQ, 4-chloro-HQ, 5,7-di-chloro-HQ, 5,7-di-bromo-HQ, and 5,7-di-iodo-HQ, were mixed with TBPI diluted in chloroform and were examined for platinum and palladium (each metal concentration 5 × 10^−4^ mol m^−3^) extraction from 5 mol of an m^−3^ hydrochloric acid solution. For platinum, a very poor distribution ratio (*D*) was noted, i.e., between 0.08 and 0.2, whereas for palladium, the highest *D* value, i.e., 230, was reported. This study found that these combinations are suitable for palladium separation from platinum^5^.

The synergistic extraction of trivalent rhodium (1.94 × 10^−4^ mol dm^−3^ metal concentration) was studied using salicylhydroamic acid (SHA) combined with the 1, 10-phenanthroline (Phen) extractant system. The extraction equilibrium time was fixed at 4 h at a temperature of 25 °C, and a feasible pH condition for positive synergistic extraction was between 6.0 and 6.5. The obtained data confirmed that the ion-exchange extraction mechanism with the release of three hydrogen ions (based on a slope analysis method) and a synergistic extraction reaction was as follows^6^:
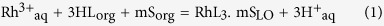


Here, HL = SHA, S = Phen, aq = aqueous phase, org = organic phase, LO = Loaded organic

Nitrogen-based calix^4^ arene was prepared and utilized for the synergistic extraction of rhodium and other elements from nitrate solutions. For the extractant 25, 26, 27, 28-etrahydroxy-5, 11, 17, 23-tetra-[4-(nhydroxyl-3-phenylprop-2-enimidamido) phenylazo] calix^4^ arene (THPAC) mixed with 30% of 1, 2-di-chloroethane and at 3.0 mol dm^−3^ nitric acid, the value of *D* was reported to be 32.68. However, at the same time, the *D* values for co-extracted metals were as follows: Cu(II)-34, Co(II)-35.42, and Ir(III)-30.6. This indicates that the separation possibilities are very narrow between associated elements^7^.

Two systems have been reported with regard to the synergistic extraction of rhodium from chloride solutions^8^. The three extractants of (di-n-hexyl sulfide (DHS), tri-n-octylamine (TOA) and N,N-dimethyl-N,N-di-n-octyl-thio-di-glycol-amide (TDGA) were diluted in chloroform and set as TDGA-TOA in one system, with another one being the DHS-TOA system for rhodium extraction. An independent TOA system does not extract rhodium metal; however, when it is paired with TDGA and DHS, the percentage extraction increases and the system shows positive synergistic behavior. Based on a slope analysis and Job’s methods, the following synergistic extraction reaction is proposed^9^:
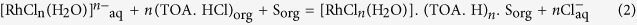


Here, n is the number, aq denotes the aqueous phase, org represents the organic phase, and S denotes TDGA or DHS.

Three mineral acids, hydrochloric, sulfuric and nitric acid solutions, were utilized for tetravalent platinum extraction with mono-thio-phosphinic acid mixed with the P-based extractants of Cyanex 272, PC 88A, D2EHPA and TBP^10^. In this study, it is important to note that the hydrochloric and sulfate solutions did not show positive extraction, whereas in the nitrate solutions, instances of positive extraction were observed (the synergistic co-efficient was between 0.161 and 0.266). In another study, three complete systems were studied, with antagonistic behavior reported (negative extraction of platinum from chloride solutions). In that work, the extractant was 0.001 mol dm^−3^ of Aliquat 336 and the synergists were 0.001 to 0.005 mol dm^−3^ of Cyanex 272, PC 88A, D2EHPA, or TBP^10^.

The present experimental study developed an extraction and separation process for precious metals such as platinum and rhodium using crown ethers as extractant systems. The four crown ethers select here are 18-crown-6 (18C6), benzo-15-crown-5(15C5), di-benzo-18-crown-6(DB18C6), and di-cyclohexano-18-crown-6 (DC18C6). For better separation between platinum and rhodium, synergistic systems are examined here. Two N-based extractants, Alamine 336 and 304-I, were mixed as a synergist with the aforementioned four CEs. The base for the four crown ethers was 15-crown-5 or 18-crown-6. The radii of the platinum and rhodium metal ions and the cavity diameters of the CEs are presented in Table 1.

## Results and Discussion

### Kinetics studies: Effect of time on platinum and rhodium extraction

The kinetics of the liquid-liquid extraction (solvent extraction) process is a function of both the kinetics of the various chemical reactions occurring in the system and the rates of diffusion of the various species that control the chemistry of the extraction process. It is important to investigate the rate at which the solute is transferred between the two phases; in some cases by an alteration of the contact time, it is possible to alter the selectivity of the extraction.

For slow kinetics, the retention time in the extraction stages must be greater than those in a system involving fast kinetics. Very fast kinetics of extraction allows for the use of contactors which have retention times on the order of seconds, thus facilitating high flow rates. Generally, metal extraction is governed by mass transfer and diffusion rates which are, on the whole, fairly rapid. As with all reactions, the slowest step in the process is the rate- determining step; that is, it controls the overall rate of the system. The formation of an extractable complex may be much slower than the rate at which the complex is extracted into the organic phase^11^:
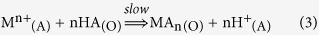


Here, M denotes the metal, HA is the extractant, A is the aqueous phase, and O indicates the organic phase.
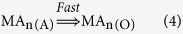


In this study, the following conditions were applied: the phase ratio (aqueous:organic) is the unit phase ratio (one), the metal concentrations were 5 × 10^−4^ mol dm^−3^ for platinum and rhodium and CE concentration is 5 × 10^−3^ mol dm^−3^. One min time is adequate for Pt extraction and 1 to 5 min is ideal for Rh extraction to reach extraction equilibrium. This shows that all of the CEs show fast kinetics reactions for the Pt/Rh extraction process.

### Hydrochloric acid effect on platinum and rhodium extraction using crown ethers

**Figure 1:**
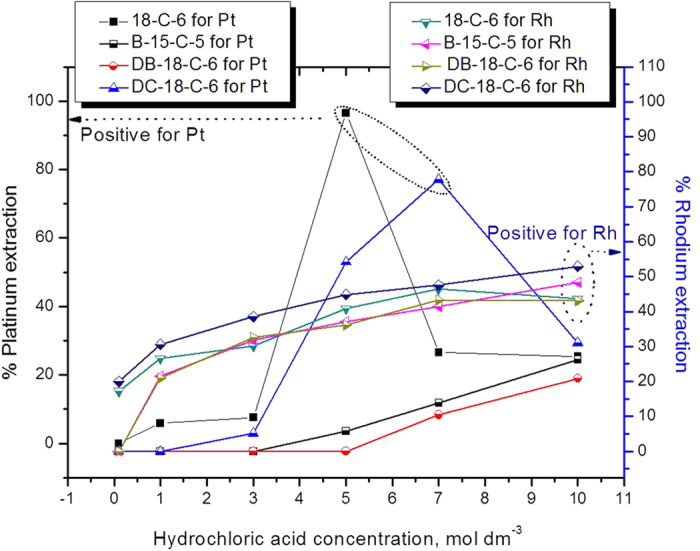
Influence of hydrochloric acid concentration on platinum and rhodium extraction using crown ethers (CE’s) as extractant systems.

The effects of an acid, specifically hydrochloric acid, on the platinum and rhodium extraction processes considering the experimental conditions were assessed. For this experiment following conditions were applied: the phase ratio (A:O) was 1, the temperature was 25 °C, and the CE concentrations in organic phase were 5 × 10^−3^ mol dm^−3^. The results obtained are presented in Fig. 1. The wide acidity range studied in the present systems is 0.1 to 10 mol dm^−3^ of hydrochloric acid concentrations. For platinum extraction, it was observed that, using 18-crown-6 (or) di-cyclohexano-18-crown-6 the extraction percentage increases with an increase in the acidity up to 5.0 mol dm^−3^ HCl (~96%) for 18-C-6 and 7 mol dm^−3^ HCl (~77%) for DC-18-C-6 and after that, it was decreased to ~25 to 31% at higher acidity such as 10 mol dm^−3^ HCl concentration. The other CEs i.e benzo-15-crown-6 (or) di-benzo-18-crown-6 showed poor extractability of platinum at studied acidity range (0 to 26%). The % of platinum extraction efficiency by using CEs order as follows: 18C6 > DC18C6 > B15C6 > DB18C6. The data obtained for rhodium extraction by CEs in same acidity range (0.1 to 10 mol dm^−3^) are presented in Fig. 1. The results indicate that three CEs show extraction percentages which increase with an increase in the acid concentration. However, this is not the case for 18-crwon-6 (18C6); at an acidity level of 7 in between 10 mol dm^−3^ of HCl concentration, the value decreases from ~46% to 43%. DC18C6 shows the highest extraction percentage of rhodium (~56%) at10 mol dm^−3^ of acidity. The extraction efficiency for rhodium by using CEs order as follows: DC18C6 > B15C6 > 18C6 > DB18C6.

The extraction behavior of platinum (or) rhodium using crown ethers as an extractant systems the following reaction mechanism was expected based on reported literature^12^:
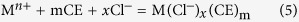


Here M = Metal (platinum (or) rhodium), CE = Crown ether, *m* or *n* or *x* = number
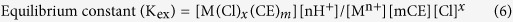


### Importance of separation factor (β)

The separation factor is one distribution ratio divided by another; it is a measure of the ability of the system to separate two solutes. For a situation in which the separation of two metals is to be done from the same solution, a useful indication as to whether this can be achieved is given by the separation process and measured by the separation factor (β). This is defined in terms of the distribution coefficients (*D*) for both metals in similar systems, as follows:
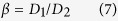


Separation factors (SFs) greater than one indicates that the two metals in question can be separated, but this gives no indication of the number of stages which may be required. Generally, with a higher the separation factor, fewer stages are required to achieve a given metal ratio in a loaded solvent^11^.

### Influence of acid concentration on separation factors of platinum and rhodium

**Figure 2:**
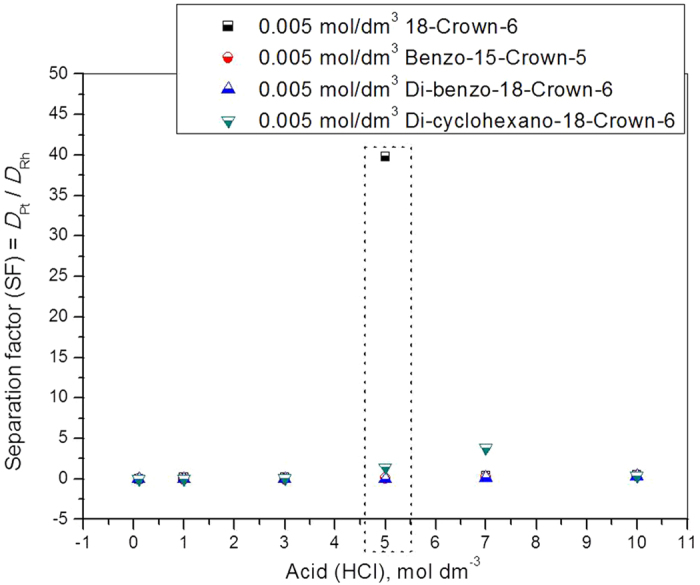
Influence of hydrochloric acid concentration on the separation factors (SF’s) of platinum and rhodium by crown ethers (CE’s) as extractant systems.

The obtained data for the separation factors (SFs) of the platinum distribution ratio divided by the rhodium distribution ratio shows that the highest SF was 0.005 mol dm^−3^ for 18-crown-6 at a hydrochloric acid concentration of 5.0 mol dm^−3^ (Fig. 2). To improve the separation potential for platinum and rhodium further, the behavior of a mixture of extractants were studied. Amine-based extractants utilized for platinum extraction and separation from rhodium, but the reported data make known that, marginal separation factors was obtained and reported^9^,^10^. Hence, the present work focused on amines mixed with CEs for maximum separation as well as the very low co-extraction of unwanted metal.

### Mixture of extractants for the platinum and rhodium extraction/separation process

Two amine-based extractants were assessed in the present study, Alamine 336 (a water-insoluble tri-octyl/decyl amine) and Alamine 304-I (a water-insoluble tri-n-dodecyl amine). Both extractants contain a nitrogen atom, and both can react with inorganic or organic acids to form an amine salt in the first step (protonation process). This amine salt participates in the ion-exchange process with other directed anions. This is described below:
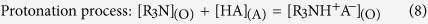


Here, [R_3_N] denotes Alamine 336 or Alamine 304-I, O is the organic phase, and A indicates the aqueous phase.
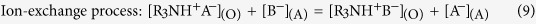


Here, [R_3_NH^+^A^−^] represents the amine salt, and [R_3_NH^+^B^−^] is the loaded amine with metal.

### Alamine 336 as an extractant and CEs as synergists

**Figure 3:**
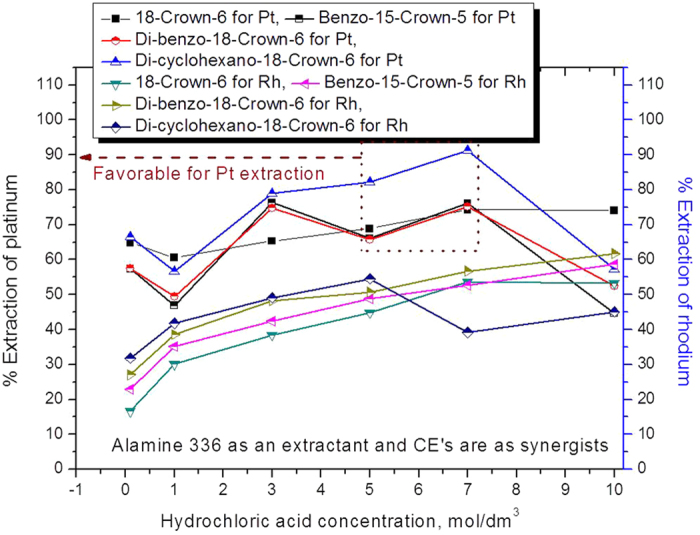
Influence of hydrochloric acid concentration on the extraction of platinum and rhodium using a mixture of extractants (Alamine 336 + CEs) (0.005 mol dm^−3^ Alamine 336 (or) CE).

The mixture of extractants was studied for the platinum and rhodium extraction/separation process. The hydrochloric acid range studied was 0.1 to 10 mol dm^−3^, and 0.005 mol dm^−3^ of Alamine 336 was mixed with 0.005 mol dm^−3^ of CEs. The obtained results are presented in Fig. 3.

The data indicate that the extraction percentage of platinum increased with the acidity up to 7.0 mol dm^−3^ and thereafter decreased, reaching 44% (by using B15C5 as a synergist). The advantage of mixing the two extractants was that all four CEs were able to extract the platinum metal at rates ranging from 57 to 91%, whereas for a single-extractant system, the two CEs of B15C5 and DB18C6 not reach an extraction efficiency of ~26%. The order of platinum extraction efficiency was as follows: di-cyclohexano-18-crown-6 + Alamine 336 > di-benzo-18-crown-6 + Alamine 336 ≥ benzo-15-crown-5 + Alamine 336 ≥ 18-crown-6 + Alamine 336.

For rhodium extraction, the four CEs were co-extracted to a minimum of 16% and a maximum of 61%. The order of rhodium extraction efficiency was as follows: di-benzo-18-crown-6 + Alamine 336 > benzo-15-crown-5 + Alamine 336 > 18-crown-6 + Alamine 336 > di-cyclohexano-18-crown-6 + Alamine 336.

### Alamine 304-I as an extractant and CEs as synergists

**Figure 4:**
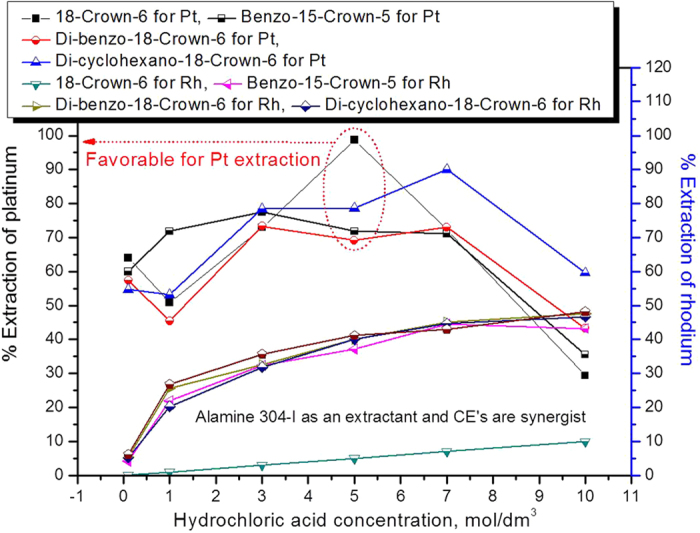
Influence of hydrochloric acid concentration on the extraction of platinum and rhodium using a mixture of extractants (Alamine 304-I + CEs) (0.005 mol dm^−3^ Alamine 304-I (or) CE).

Alamine 304-I mixed with the four CEs of 18-crown-6, benzo-15-crown-5, di-benzo-18-crown-6 and di-cyclohexano-18-crown-6, and both the extractant and synergist concentrations were kept constant at 0.005 mol dm^−3^. The obtained data is presented in Fig. 4.

Platinum extraction achieved a maximum rate of 98.7% with 18-crown-6 as a synergist at a hydrochloric acid concentration of 5.0 mol dm^−3^. On the other hand, with the other three CEs, platinum was extracted at rates between 54 and 78% by mixing with Alamine 304-I. Except for 18-crown-6, the other CEs showed improved extraction efficiency of 48% to 78% in the presence of Alamine 304-I. At a particular acid point, such as an HCl concentration of 5 mol dm^−3^, the order of platinum extraction efficiency was as follows: 18-crown-6 + Alamine 304-I > di-cyclohexano-18-crown-6+ Alamine 304-I > benzo-15-crown-5 + Alamine 304-I > di-benzo-18-crown-6 + Alamine 304-I, whereas in the case of rhodium, the mixture of extractants influenced that all cases of extraction enhancement except for 0.005 mol dm^−3^ of di-cyclohexano-18-crown-6 as a synergist at a high acidity level of 7.0 mol dm^−3^ (Fig. 4).

### Role of the separation factor in platinum and rhodium extraction according to the mixture of extractants

**Figure 5:**
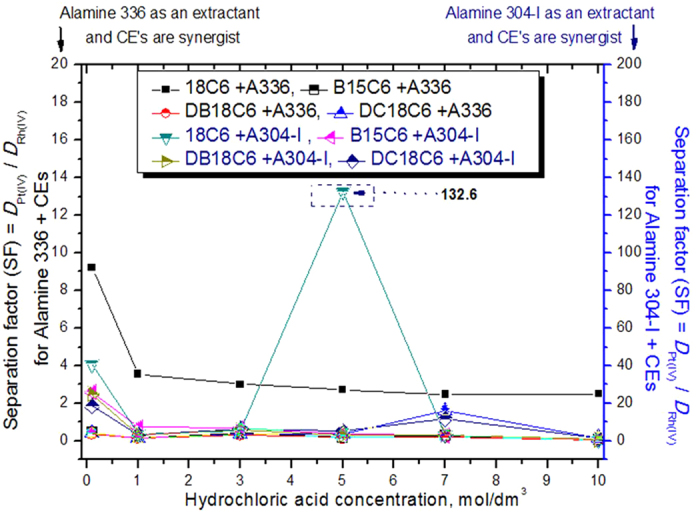
Influence of hydrochloric acid concentration on platinum and rhodium extraction using Alamine 336 (or) Alamine 304-I as extractant systems mixed with CE’s as synergists.

Both amine-based extractants (Alamine 336 and Alamine 304-I) were mixed with each CE and positive extraction data was determined for both metals. The aim of the present mixture of extractants was to improve the separation potential of both metals. The obtained extraction distribution data, i.e., the calculated separation factors, are presented in Fig. 5.

For Alamine 336 as an extractant system and CEs as synergists, the SF did not reach 10. The highest SF was observed at a lower acidity level of 0.1 mol dm^−3^ HCl with 18C6 mixed with Alamine 336. The next highest acidity level of 7.0 mol dm^−3^ HCl with DC-18C6 shows better SF between Pt and Rh, whereas without Alamine 336, the single CEs were able to reach a SF of 11.5. On the other hand, with Alamine 304-I as an extractant system and CEs as synergists, the highest SF of 132.6 was calculated between Pt and Rh at an HCl concentration of 5 mol dm^−3^. This study concludes that Alamine 304-I is better than Alamine 336 when combined with CEs for the separation of Pt and Rh.

### Synergism and antagonism theory

A mixture of two different types of extractants (E), i.e., extractant 1 (E1) and extractant 2 (E2), can enrich the extraction of metal ions under certain optimum experimental conditions, such as an optimum temperature, time, pH and metal ion concentration. The occurrence of a synergic effect is usually connected to a process that arises on account of the complexity of the organic phase. The organic phase may contain an extractant and a diluent. The extractant and diluent form a phase immiscible with water. The extractant and diluent may react with metal ions to yield compounds soluble in the organic phase^11^.

The variety of systems in which synergism has been observed indicates that the extraction mechanism of the synergistic process is not indistinguishable in all cases. However, synergism which occurs in the organic phase may be caused by one of the following factors: 1) the activity of the extractant is affected thermodynamically, or 2) the composition of the metal-bearing species forms in the organic phase is not identical to that in the case of a single-extractant system. In general, synergistic systems are classified into four types: 1) acidic extractant + neutral synergist, 2) neutral extractant + acidic synergist, 3) neutral extractant + neutral synergist, 4) chelating extractant + chelating synergist^13^. The magnitude of synergism can be represented in terms of synergistic enhancement factor, defined as:
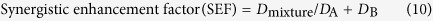


To quantify the synergic effect, the synergic coefficient (S.C.) is defined as follows:
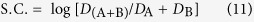
Here, *D*_A_ is the distribution ratio of the extractant, *D*_B_ denotes the distribution ratio of the synergist, *D*_(A+B)_ is the distribution ratio of the mixture (the extractant mixed with the synergist).

If S.C. is >0, a positive synergic effect is said to occur; if there is an antagonistic effect (negative synergism), S.C. < 0, and if there is no synergic effect, S.C. = 0.

The nature and extent of the synergic effect depend primarily on the properties of the synergist itself, but the properties of the metal, the diluent and the nature of the chelate-forming reagent can also contribute to this effect. The formation of mixed complexes is believed to be the main cause of the synergic effect. It can be demonstrated that if the molecules of a neutral donor (synergist) have removed water molecules from an extractable chelate, the complex becomes less hydrophilic; thus, the extraction is improved^11^.

### Synergistic studies with platinum: Alamine 336 or Alamine 304-I as an extractant system and CEs as synergists

Synergistic extraction studies were conducted for platinum extraction using Alamine 336 or Alamine 304-I as an extractant system mixed with four different CEs (18-crown-6, benzo-15-crown-5, di-benzo-18-crown-6, or di-cyclohexano-18-crown-6) under optimum experimental conditions, i.e., an optimum extraction time and a wide acid range. The acid range used here was 0.1 to 10 mol dm^−3^, and both extractant concentrations were fixed at 5 × 10^−3^ mol dm^−3^. The phase ratio is the unit phase ratio, the extraction time was 1 min for the Alamine 336 systems and 10 to 15 min for the Alamine 304-I systems, and the experiments were carried out at a temperature of 25 °C. The calculated synergistic enhancement factor (S.E.F) of the platinum is presented in Table 2 from the CEs + Alamine 336 systems. Table 3 shows this data CEs + Alamine 304-I systems.

#### Alamine 336 as an extractant system

For the first synergist, 18-crown-6, the synergetic extraction behavior was as follows: a lower acid range of 0.1 to 1.0 mol dm^−3^ and a higher acid range of 7.0 to 10.0 mol dm^−3^ of hydrochloric acid were favorable for the enhanced extraction of platinum, whereas with 5.0 mol dm^−3^ of hydrochloric acid, negligible enhancement was noted. In the case of benzo-15-crown-5 as a synergist, the entire acid range of 0.1 to 7.0 mol dm^−3^ of hydrochloric acid showed positive enhancement, except at the higher acidity of 10.0 mol dm^−3^ of hydrochloric acid. On the other hand, di-benzo-18-crown-6 or di-cyclohexano-18-crown-6 as synergists showed a total range of acidity of 0.1 to 10.0 mol dm^−3^, thus demonstrating promising extraction enhancement.

#### Alamine 304-I as an extractant system

Positive extraction enhancement was observed with 18-crown-6 or benzo-15-crown-5 as a synergist in the entire acid range. In the case of 18C6, highest SEF values around 2.65 were observed at 5.0 mol dm^−3^ of hydrochloric acid concentration, whereas for the B15C6 system, the highest SEF values around 2.886 at 1.0 of hydrochloric acid concentration. In the case of di-benzo-18-crown-6 as a synergist, except at an acidity level of 0.1 mol dm^−3^ (SEF = 1.86), all SEF values were around 1 ± 0.1. The fourth CE i.e., di-cyclohexano-18-crown-6 as a synergist, showed advantageous extraction enhancement entire the range. Overall, all four CEs showed auspicious extraction enhancement for the platinum extraction process.

**Figure 6:**
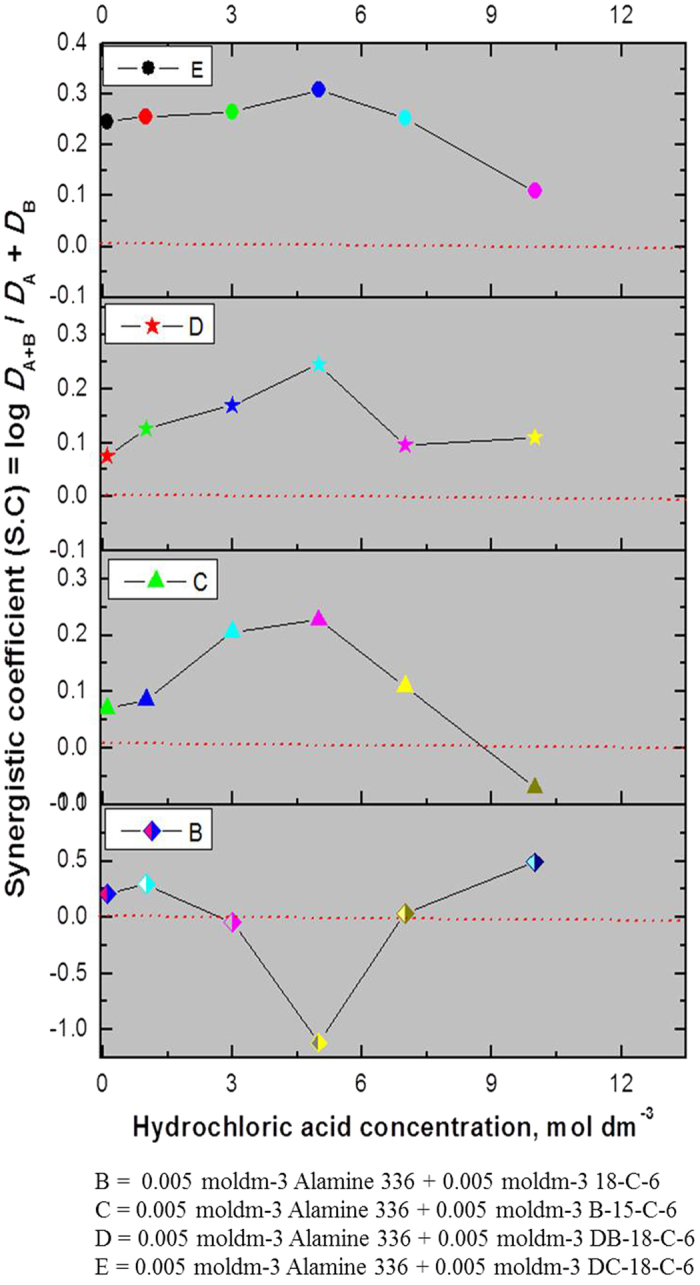
Synergistic studies for platinum: Alamine 336 mixed with CE’s [Plot in between hydrochloric acid concentration vs synergistic co-efficient (S.C)].

**Figure 7:**
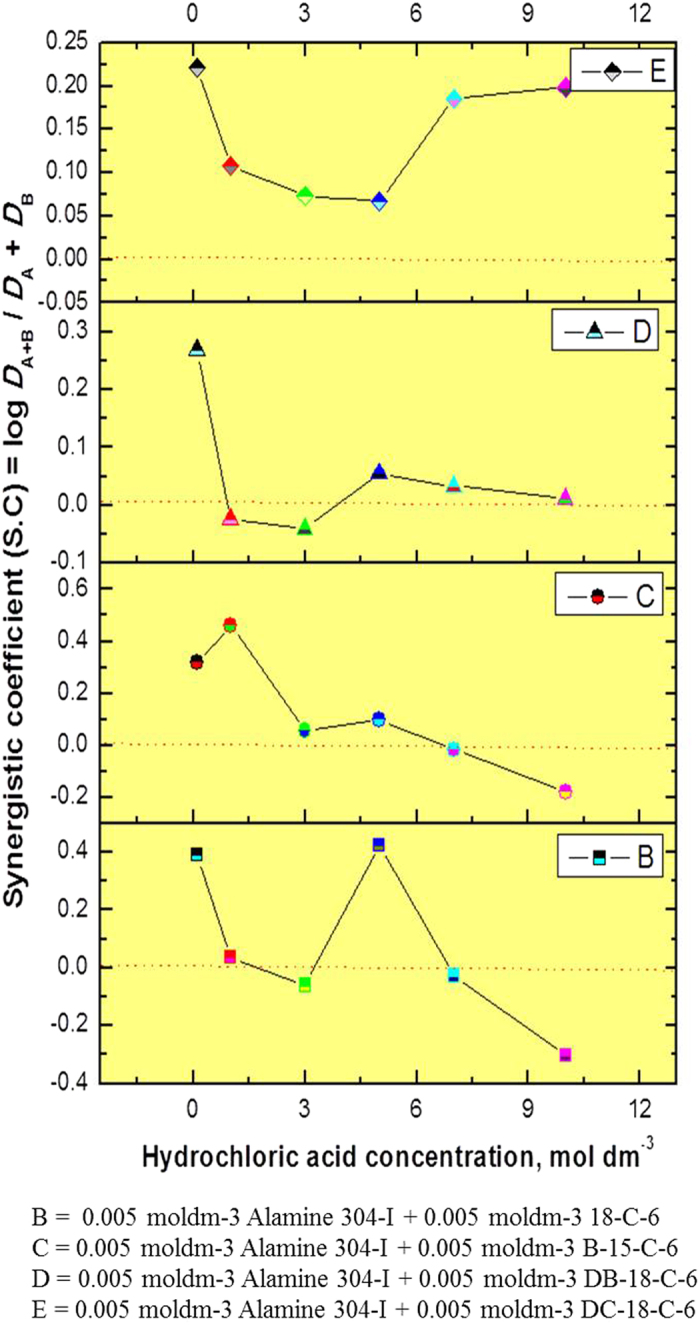
Synergistic studies for platinum: Alamine 304-I mixed with CE’s [Plot in between hydrochloric acid concentration vs synergistic co-efficient (S.C)].

The synergistic coefficient (S.C.) data with all CEs mixed with Alamine 336 or Alamine 304-I is presented in Figs 6 and 7. S.C. values of ‘0’ or above indicate synergistic behavior, while those below ‘0’ show anti-synergistic behavior, indicative of antagonism. In the first case of the alamine 336 + CEs systems: 18C6 as a synergist in an acidity range of 3.0 to 5.0 mol dm^−3^ resulted in negative synergism. In the other case, i.e., the alamine 304-I + CEs systems: 18C6 as a synergist showed lower acidity (below 1 mol dm^−3^), indicative of positive synergism, while at a higher acidity level of 5.0 mol dm^−3^, an HCl synergistic effect was observed. B15C5 as a synergist at an acidity level of 10 mol dm^−3^ shows negative synergism. In the case of DB18C6 apart from lower acidity levels (below 1 mol dm^−3^), the remaining acid range did not show a meaningful effect, while the HCl range of 1 to 3 mol dm^−3^ showed a negative effect. For the other two CEs with DC18C6, the entire acid range from 0.1 to 10 mol dm^−3^ shows positive synergistic behavior.

### Synergistic studies for rhodium: Alamine 336 or Alamine 304-I as an extractant system and CEs as synergists

**Figure 8:**
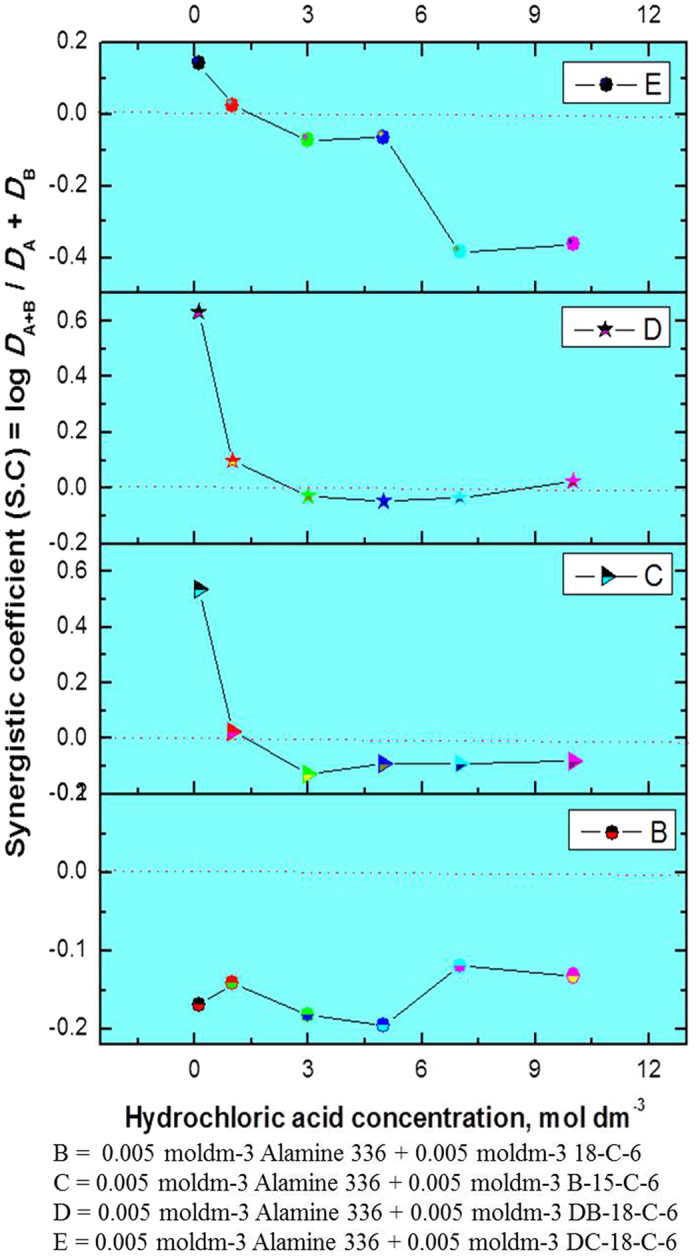
Synergistic studies for rhodium: Alamine 336 mixed with CE’s [Plot in between hydrochloric acid concentration vs synergistic co-efficient (S.C)].

**Figure 9:**
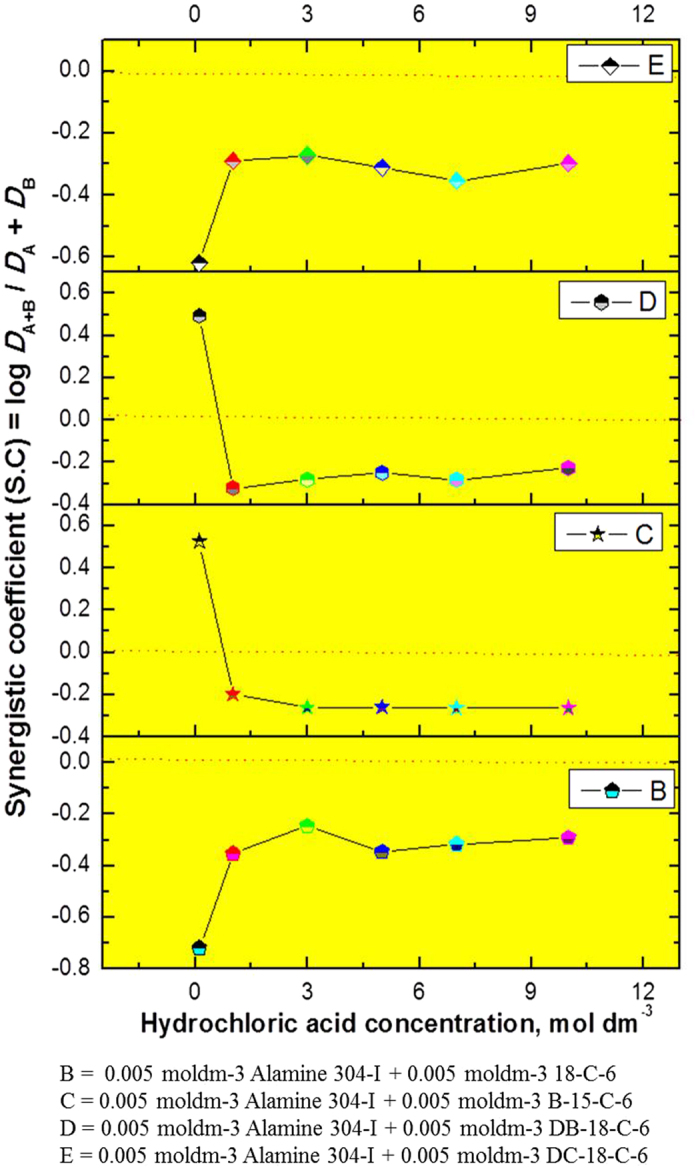
Synergistic studies for rhodium: Alamine 304-I mixed with CE’s [Plot in between hydrochloric acid concentration vs synergistic co-efficient (S.C)].

The synergistic enhancement (SEF) of rhodium using Alamine 336 or Alamine 304-I as an extractant system mixed with CEs as synergistic systems was studied, and the results obtained are presented in Tables 4 and 5. The synergistic coefficient (S.C.) data with the all CEs are presented in Figs 8 and 9. Here, the acidic range was 0.1 to 10 mol dm^−3^, and both extractant concentrations were fixed at 5 × 10^−3^ mol dm^−3^. In addition, the ratio is the unit phase ratio, and the experiments were carried out at a temperature of 25 °C.

S.C. values of ‘0’ or above show synergistic behavior. Those below ‘0’, showing anti-synergistic behavior, indicate antagonism. For alamine 336 mixed with the CEs, in all four systems, the HCl acid concentration range is 1.0 to 10 mol dm^−3^, showing negative synergism. In contrast, at lower acidity levels such as 0.1 mol dm^−3^ HCl, a highly synergistic effect was observed for three CEs (B15C5, DB18C6 and DC18C6). This is a good indication that for the CEs, possible separation of both metals will be increased by a mixture of extractants. Nearly parallel behavior was observed for the alamine 304-I + CEs systems; apart from the B15C6 and DB18C6 combinations, all showed positive synergistic behavior at the lower acidity level of 0.1 mol dm^−3^ HCl for rhodium.

The synergistic extraction behavior of platinum (or) rhodium using amines mixed with crown ethers as an extractant system the following reaction mechanism was expected based on reported literature^12^,^14^,^15^:
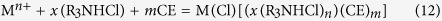


Here M = Metal (Pt (or) Rh), *x* or *m* or *n* = 1, 2, 3.. etc, R_3_N = Alamine 336 (or) Alamine 308, CE = Crown ether
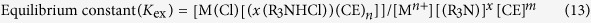


## Materials and Methods

### Apparatus and reagents

Aqueous solutions (feed and raffinate solutions) of metal ion concentrations were analyzed using an inductively coupled plasma optical emission spectrometer (ICP-OES) (manufactured by Thermo Scientific, USA, model iCAP 6000 Series). Platinum and rhodium were determined by the following wavelengths (nm): platinum = 214.423 and rhodium = 343.489. The ICP conditions are an Ar gas pressure of 80 psi, RF power of 1350 w, a pump rate of 40 rpm, an auxiliary gas flow rate of 0.5 L/min, a Neb. gas flow rate of 0.5 L/min, a chiller temperature of 24.1 °C, a camera temperature of −46.55 °C, a generator temperature of 31 °C, and an optics temperature of 38.1 °C.

A shaking incubator (Model: SI-300/300R/600/600R) was used in the solvent extraction step and in the synergistic solvent extraction experiments. Kerosene (boiling point: 180–270 °C, density: 0.80) was used as a diluent in the system. This study utilized the amine-based extractants of Alamine 336 (tri-octyl/decyl amine) and Alamine 304-I (tri-n-dodecyl amine), supplied by Cognis, USA.

**Figure 10:**
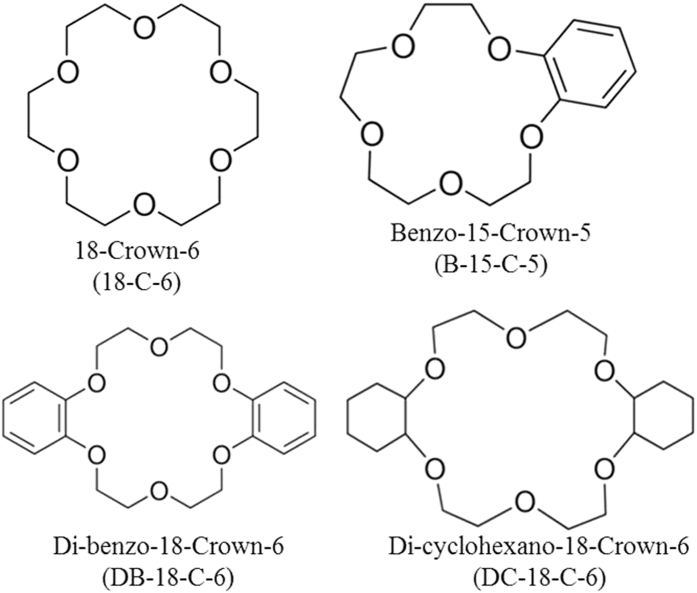
Molecular structures of four crown ethers used in present study (Adapted from Aldrich Chemicals).

Stock solutions of platinum and rhodium metal ions were prepared by dissolving appropriate corresponding amounts of the metal salts H_2_PtCl_6_.nH_2_O and RhCl_3_.3H_2_O in distilled water. The initial metal ion concentrations were 5 × 10^−3^ mol dm^−3^ in both cases for all extraction and synergistic extraction studies. All organic phases were prepared by dissolving weighed amounts of 18-crown-6 (18C6), benzo-15-crown-5 (15C5), di-benzo-18-crown-6 (DB18C6), di-cyclohexano-18-crown-6 (DC18C6), Alamine 336 and Alamine 304-I in kerosene diluted to the require volumes. The four CEs supplied by Aldrich, USA, and the molecular structures of the CEs are presented in Fig. 10.

### Extraction procedure

The distribution ratios (*D*) of the metal ions (platinum or rhodium) from the aqueous phase to the organic phase for times of 1 min (for CEs mixed with Alamine 336) and 10 to 15 min (for CEs mixed with Alamine 304-I) in a glass-stoppered separating funnel were determined after reaching extraction equilibrium. All extraction and synergistic extraction experiments were carried out at a temperature of 25 ± 1 °C and a shaker speed of 250 rpm. After extraction/synergistic extraction, the solutions were allowed to settle, and the organic and aqueous phases were separated and evaluated using an inductively coupled plasma optical emission spectrometer (ICP-OES). The raffinate solution used here indicates the remaining metal ion concentrations after the extraction/synergistic extraction process. The concentrations of the metal ions in the organic phase were then obtained through the material balance. These concentrations were utilized to measure the metal distribution ratios from the aqueous phase to the organic phase during the extraction process. Good general agreement was obtained, with distribution ratio values within ± 3% in all cases.

The present study mainly used a major formula, i.e., the percentage extraction of the metal and the metal distribution between two phases.
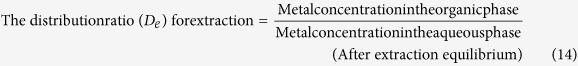


Here, the metal is either platinum (or) rhodium
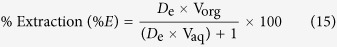


Here, V_org_ = Volume of organic phase, V_aq_ = Volume of aqueous phase.

## Conclusion

Macrocyclic compounds such as crown ethers, having specific cavity diameters, offer better extraction and the possible separation of difficult metal groups. In the present study, we proposed a new means of extraction separation for the two PGM metals of platinum and rhodium. Independent CEs were used to extract both of these metals. The maximum separation factor was calculated and found to be approximately 39.8 at a higher acidity of HCl (5.0 mol dm^−3^) using 0.005 mol dm^−3^ 18-crown-6 as an extractant system. Two nitrogen-based extractants, Alamine 336 and Alamine 304-I, were utilized as mixed extractant systems with all four CEs. The highest separation factor of 132.6 was found using the 18-crown-6 + Alamine 304-I system at 5.0 mol dm^−3^ HCl.

The second target of this study was to test for possible synergistic extraction enhancements with these mixtures. The synergistic enhancement factor (SEF) of platinum when using the Alamine 336 + CEs (18-crown-6 or benzo-15-crown-5 or di-benzo-18-crown-6 or di-cyclohexano-18-crown-6) mixtures indicated very fast kinetics (reaching extraction equilibrium within one minute), and the highest SEF value was obtained with Alamine 336 + 18-crown-6, i.e., ~3 at a higher acidity level (10 mol dm^−3^ HCl). The order of the SEF with these mixtures was as follows: (Alamine 336 + 18C6) >(Alamine 336 + DC18C6) >(Alamine 336 + DB18C6) >Alamine 336 + B15C6). Whereas in the case of Alamine 304-I mixed with CEs, lower acidity was most favorable, at 0.1 to 1.0 mol dm^−3^ HCl; the highest SEF values were 2.47 for the Alamine 336 + 18C6 system and 2.886 for the Alamine 336 + B15C6 system. In the case of rhodium, Alamine 336 mixed with CEs resulted in the highest SEF values of 4.3 (Alamine 336 + B15C6) and 3.44 (Alamine 336 + DB18C6) at 0.1 mol dm^−3^ HCl, indicating poor separation potential between platinum and rhodium. An identical system combining Alamine 304-I for rhodium at the same acidity level showed the highest SEF values of 3.352 and 3.117.

This study concludes that better separation and good synergistic enchantment during the extraction of both metal ions were developed. To the best of our knowledge, using CEs combined with N based extractant systems platinum and rhodium extraction and possible separation was not reported yet in the literature.

## Additional Information

**How to cite this article**: Jyothi, R.K. and Lee, J.-Y. The role of macrocyclic compounds in the extraction and possible separation of platinum and rhodium from chloride solutions. *Sci. Rep.* **6**, 27668; doi: 10.1038/srep27668 (2016).

## Figures and Tables

**Table 1:** The present study utilized metal ions radii and crown ethers cavity diameters, *d*.

Metal salt^16^	Metal ion	*d*/*A*^*o*^^17^	Crown ether	*d*/*A*^*o*^^18^
H_2_PtCl_6_.nH_2_O	Pt^4+^	1.25	18-Crown-6	1.34–1.43
			Benzo-15-Crown-5	1.72–1.84
RhCl_3_.3H_2_O	Rh^3+^	1.33	Di-benzo-18-Crown-6	2.68–2.86
			Di-cyclohexano-18-Crown-6	2.68–2.86

**Table 2:** Synergistic enhancement factor of platinum by using Alamine 336 + CE’s mixture (18-crown-6 = 18C6, Benzo-15-crown-5 = B15C6, Di-benzo-18-crown-6 = DB18C6, Di-cyclohexano-18-crown-6 = DC18C6), Here E = Extractant, S = Synergist, Extraction time: 1 min.

HCl, mol.dm^−3^	Extractant (E), 5 × 10^−3^ mol dm^−3^	*D* of the extractant (*D*_A_)	Synergist (S), 5 × 10^−3^ mol dm^−3^	*D* of the synergist (S) (*D*_B_)	*D* of the mixture of E & S (*D*_A+B_)	Sum of the *D* of E & S (*D*_A_ + *D*_B_)	Δ*D = D*_A+B_− (*D*_A_ + *D*_B_)	S.E.F = *D*_A+B_/*D*_A_ + *D*_B_
0.1	Alamine 336	1.13	18C6	0	1.83	1.13	0.7	1.619
1.0	Alamine 336	0.72	18C6	0.062	1.54	0.782	0.758	1.969
3.0	Alamine 336	1.99	18C6	0.081	1.88	2.071	−0.19	0.907
5.0	Alamine 336	1.09	18C6	27.46	2.21	28.55	−26.3	0.077
7.0	Alamine 336	2.29	18C6	0.36	2.88	2.65	0.23	1.086
10.0	Alamine 336	0.59	18C6	0.33	2.85	0.92	1.93	3.097
0.1	Alamine 336	1.13	B15C6	0	1.34	1.13	0.21	1.18
1.0	Alamine 336	0.72	B15C6	0	0.88	0.72	0.16	1.22
3.0	Alamine 336	1.99	B15C6	0	3.22	1.99	1.23	1.61
5.0	Alamine 336	1.09	B15C6	0.06	1.95	1.15	0.8	1.69
7.0	Alamine 336	2.29	B15C6	0.16	3.18	2.45	0.73	1.29
10.0	Alamine 336	0.59	B15C6	0.35	0.80	0.94	−0.14	0.85
0.1	Alamine 336	1.13	DB18C6	0	1.35	1.13	0.22	1.19
1.0	Alamine 336	0.72	DB18C6	0	0.97	0.72	0.25	1.34
3.0	Alamine 336	1.99	DB18C6	0	2.95	1.99	0.96	1.48
5.0	Alamine 336	1.09	DB18C6	0	1.92	1.09	0.83	1.76
7.0	Alamine 336	2.29	DB18C6	0.11	3.01	2.4	0.61	1.25
10.0	Alamine 336	0.59	DB18C6	0.26	1.10	0.85	0.25	1.29
0.1	Alamine 336	1.13	DC18C6	0	1.99	1.13	0.86	1.761
1.0	Alamine 336	0.72	DC18C6	0	1.30	0.72	0.58	1.805
3.0	Alamine 336	1.99	DC18C6	0.054	3.76	2.04	1.72	1.843
5.0	Alamine 336	1.09	DC18C6	1.18	4.63	2.27	2.36	2.039
7.0	Alamine 336	2.29	DC18C6	3.48	10.34	5.77	4.57	1.792
10.0	Alamine 336	0.59	DC18C6	0.45	1.34	1.04	0.30	1.288

**Table 3:** Synergistic enhancement factor of platinum by using Alamine 304-I + CE’s mixture (18-crown-6 = 18C6, Benzo-15-crown-5 = B15C6, Di-benzo-18-crown-6 = DB18C6, Di-cyclohexano-18-crown-6 = DC18C6), Here E = Extractant, S = Synergist, Extraction time: 10 to 15 min.

HCl, mol.dm^−3^	Extractant (E), 5 × 10^−3^ mol dm^−3^	*D* of the extractant (*D*_A_)	Synergist (S), 5 × 10^−3^ mol dm^−3^	*D* of the synergist (S) (*D*_B_)	*D* of the mixture of E & S (*D*_A+B_)	Sum of the *D* of E & S (*D*_A_ + *D*_B_)	Δ*D = D*_A+B_− (*D*_A_ + *D*_B_)	S.E.F = *D*_A+B_/*D*_A_ + *D*_B_
0.1	Alamine 304-I	0.72	18C6	0	1.78	0.72	1.06	2.47
1.0	Alamine 304-I	0.88	18C6	0.062	1.03	0.942	0.088	1.09
3.0	Alamine 304-I	3.02	18C6	0.081	2.70	3.101	−0.401	0.87
5.0	Alamine 304-I	1.97	18C6	27.46	78.28	29.43	48.85	2.65
7.0	Alamine 304-I	2.39	18C6	0.36	2.61	2.75	−0.14	0.94
10.0	Alamine 304-I	0.48	18C6	0.33	0.41	0.81	−0.4	0.50
0.1	Alamine 304-I	0.72	B15C6	0	1.50	0.72	0.78	2.083
1.0	Alamine 304-I	0.88	B15C6	0	2.54	0.88	1.66	2.886
3.0	Alamine 304-I	3.02	B15C6	0	3.44	3.02	0.42	1.139
5.0	Alamine 304-I	1.97	B15C6	0.06	2.54	2.03	0.51	1.251
7.0	Alamine 304-I	2.39	B15C6	0.16	2.45	2.55	−0.1	0.960
10.0	Alamine 304-I	0.48	B15C6	0.35	0.55	0.83	−0.28	0.662
0.1	Alamine 304-I	0.72	DB18C6	0	1.34	0.72	0.62	1.861
1.0	Alamine 304-I	0.88	DB18C6	0	0.83	0.88	−0.05	0.943
3.0	Alamine 304-I	3.02	DB18C6	0	2.75	3.02	−0.27	0.910
5.0	Alamine 304-I	1.97	DB18C6	0	2.24	1.97	0.27	1.137
7.0	Alamine 304-I	2.39	DB18C6	0.11	2.70	2.5	0.2	1.08
10.0	Alamine 304-I	0.48	DB18C6	0.26	0.76	0.74	0.02	1.027
0.1	Alamine 304-I	0.72	DC18C6	0	1.20	0.72	0.48	1.666
1.0	Alamine 304-I	0.88	DC18C6	0	1.13	0.88	0.25	1.284
3.0	Alamine 304-I	3.02	DC18C6	0.054	3.64	3.074	0.566	1.184
5.0	Alamine 304-I	1.97	DC18C6	1.18	3.68	3.15	0.53	1.168
7.0	Alamine 304-I	2.39	DC18C6	3.48	9.00	5.87	3.13	1.533
10.0	Alamine 304-I	0.48	DC18C6	0.45	1.47	0.93	0.54	1.580

**Table 4:** Synergistic enhancement factor of rhodium by using Alamine 336 + CE’s mixture (18-crown-6 = 18C6, Benzo-15-crown-5 = B15C6, Di-benzo-18-crown-6 = DB18C6, Di-cyclohexano-18-crown-6 = DC18C6), Here E = Extractant, S = Synergist, Extraction time: 1 min.

HCl, mol.dm^−3^	Extractant (E), 5 × 10^−3^ mol dm^−3^	*D* of the extractant (*D*_A_)	Synergist (S), 5 × 10^−3^ mol dm^−3^	*D* of the synergist (S) (*D*_B_)	*D* of the mixture of E & S (*D*_A+B_)	Sum of the *D* of E & S (*D*_A_ + *D*_B_)	Δ*D = D*_A+B_− (*D*_A_ + *D*_B_)	S.E.F = *D*_A+B_/*D*_A_ + *D*_B_
0.1	Alamine 336	0.086	18C6	0.208	0.199	0.294	−0.09	0.676
1.0	Alamine 336	0.235	18C6	0.361	0.430	0.596	−0.16	0.721
3.0	Alamine 336	0.516	18C6	0.430	0.623	0.946	−0.32	0.658
5.0	Alamine 336	0.582	18C6	0.689	0.811	1.271	−0.46	0.638
7.0	Alamine 336	0.657	18C6	0.865	1.155	1.522	−0.36	0.758
10.0	Alamine 336	0.773	18C6	0.773	1.140	1.546	−0.40	0.737
0.1	Alamine 336	0.086	B15C6	0	0.296	0.086	0.21	3.44
1.0	Alamine 336	0.235	B15C6	0.275	0.543	0.51	0.033	1.064
3.0	Alamine 336	0.516	B15C6	0.467	0.736	0.983	−0.247	0.748
5.0	Alamine 336	0.582	B15C6	0.590	0.953	1.172	−0.219	0.813
7.0	Alamine 336	0.657	B15C6	0.708	1.110	1.365	−0.255	0.813
10.0	Alamine 336	0.773	B15C6	0.936	1.420	1.709	−0.289	0.830
0.1	Alamine 336	0.086	DB18C6	0	0.370	0.086	0.284	4.302
1.0	Alamine 336	0.235	DB18C6	0.265	0.627	0.5	0.127	1.254
3.0	Alamine 336	0.516	DB18C6	0.484	0.934	1	−0.066	0.934
5.0	Alamine 336	0.582	DB18C6	0.566	1.029	1.148	−0.119	0.896
7.0	Alamine 336	0.657	DB18C6	0.763	1.310	1.42	−0.11	0.922
10.0	Alamine 336	0.773	DB18C6	0.757	1.618	1.53	0.088	1.057
0.1	Alamine 336	0.086	DC18C6	0.250	0.467	0.336	0.131	1.389
1.0	Alamine 336	0.235	DC18C6	0.440	0.717	0.675	0.042	1.062
3.0	Alamine 336	0.516	DC18C6	0.627	0.965	1.143	−0.178	0.844
5.0	Alamine 336	0.582	DC18C6	0.811	1.200	1.393	−0.193	0.861
7.0	Alamine 336	0.657	DC18C6	0.906	0.644	1.563	−0.919	0.412
10.0	Alamine 336	0.773	DC18C6	1.125	0.820	1.898	−1.078	0.432

**Table 5:** Synergistic enhancement factor of rhodium by using Alamine 304-I + CE’s mixture (18-crown-6 = 18C6, Benzo-15-crown-5 = B15C6, Di-benzo-18-crown-6 = DB18C6, Di-cyclohexano-18-crown-6 = DC18C6), Here E = Extractant, S = Synergist, Extraction time: 10 to 15 min.

HCl, mol.dm^−3^	Extractant (E), 5 × 10^−3^ mol dm^−3^	*D* of the extractant (*D*_A_)	Synergist (S), 5 × 10^−3^ mol dm^−3^	*D* of the synergist (S) (*D*_B_)	*D* of the mixture of E & S (*D*_A+B_)	Sum of the *D* of E & S (*D*_A_ + *D*_B_)	Δ*D = D*_A+B_− (*D*_A_ + *D*_B_)	S.E.F = *D*_A+B_/*D*_A_ + *D*_B_
0.1	Alamine 304-I	0.017	18C6	0.208	0.043	0.225	−0.18	0.191
1.0	Alamine 304-I	0.270	18C6	0.361	0.280	0.631	−0.35	0.443
3.0	Alamine 304-I	0.410	18C6	0.430	0.477	0.84	−0.36	0.567
5.0	Alamine 304-I	0.619	18C6	0.689	0.590	1.308	−0.71	0.451
7.0	Alamine 304-I	0.794	18C6	0.865	0.799	1.659	−0.86	0.481
10.0	Alamine 304-I	0.723	18C6	0.773	0.763	1.496	−0.73	0.510
0.1	Alamine 304-I	0.017	B15C6	0	0.057	0.017	0.04	3.352
1.0	Alamine 304-I	0.270	B15C6	0.275	0.344	0.545	−0.20	0.631
3.0	Alamine 304-I	0.410	B15C6	0.467	0.481	0.877	−0.39	0.548
5.0	Alamine 304-I	0.619	B15C6	0.590	0.666	1.209	−0.54	0.550
7.0	Alamine 304-I	0.794	B15C6	0.708	0.820	1.502	−0.68	0.545
10.0	Alamine 304-I	0.723	B15C6	0.936	0.906	1.659	−0.75	0.546
0.1	Alamine 304-I	0.017	DB18C6	0	0.053	0.017	0.036	3.117
1.0	Alamine 304-I	0.270	DB18C6	0.265	0.255	0.535	−0.28	0.476
3.0	Alamine 304-I	0.410	DB18C6	0.484	0.467	0.894	−0.42	0.522
5.0	Alamine 304-I	0.619	DB18C6	0.566	0.666	1.185	−0.51	0.562
7.0	Alamine 304-I	0.794	DB18C6	0.763	0.810	1.557	−0.74	0.520
10.0	Alamine 304-I	0.723	DB18C6	0.757	0.876	1.48	−0.60	0.591
0.1	Alamine 304-I	0.017	DC18C6	0.250	0.064	0.267	−0.20	0.239
1.0	Alamine 304-I	0.270	DC18C6	0.440	0.364	0.71	−0.34	0.512
3.0	Alamine 304-I	0.410	DC18C6	0.627	0.554	1.037	−0.48	0.534
5.0	Alamine 304-I	0.619	DC18C6	0.811	0.698	1.43	−0.73	0.488
7.0	Alamine 304-I	0.794	DC18C6	0.906	0.752	1.70	−0.94	0.442
10.0	Alamine 304-I	0.723	DC18C6	1.125	0.930	1.848	−0.91	0.503
